# Vitamin D deficiency in Ukraine: current evidence

**DOI:** 10.1186/s40795-023-00706-z

**Published:** 2023-03-14

**Authors:** N. V. Grygorieva, T. Yu. Solonenko, A. S. Musiienko, M. A. Bystrytska

**Affiliations:** Department of Clinical Physiology and Pathology of Musculoskeletal System, State Institution “D. F. Chebotarev Institute of Gerontology of the NAMS of Ukraine”, Kiev, Ukraine

**Keywords:** Ukraine, Vitamin D deficiency, Vitamin D insufficiency, 25(OH)D, Epidemiology, Age, Sex, Season

## Abstract

**Background:**

Data from numerous studies demonstrate the high frequency of vitamin D deficiency (VDD) and insufficiency (VDI) in many countries worldwide that depend on age and sex, seasons, country, and concomitant pathology. This research aimed to study vitamin D status in the Ukrainian population during 2016–2022 years depending on age, sex, month, and year of the observation, and compare the results with the data of previous Ukrainian epidemiologic studies.

**Methods:**

In a single-center cohort study, we analyzed the serum total 25-hydroxyvitamin D (25(OH)D) level in 7,418 subjects aged 20–99 years. The analysis was performed depending on age, sex, month, season, and year of the observation. Also, we compared the results with the data of previous Ukrainian studies. 25(OH)D level was measured using the electrochemiluminescence method.

**Results:**

The mean serum 25(OH)D level in the total group was 31.0 [22.3–41.1] ng/mL, the lowest level was in the age group 90–99 years old. No gender differences were found in 25(OH)D levels, except the one for the women aged 60–69 years old who had higher vitamin D levels compared to male parameters. 41.6% of the subjects had an optimal (> 30–50 ng/mL) 25(OH)D level, 27.3% had VDI, and 19.5% had a VDD. The suboptimal and high serum concentration of 25(OH)D (> 50–100 ng/mL) was found in 11.4% of the subjects. Also, we established the increase of serum 25(OH)D level from 2016 to 2022 with the highest values in 2020–2022. Seasonal variations of 25(OH)D concentration confirmed the highest index in autumn (33.0 [24.0–42.4] ng/mL) and the lowest one in the spring (28.5 [19.7–38.7] ng/mL) with the highest 25(OH)D level in September and the lowest one in March.

**Conclusion:**

Our results confirmed a decrease in VDD and VDI in 2020–2022 in the Ukrainian population compared to the previous years (2016–2019) and previous Ukrainian studies with similar age and seasonal particularities. It may be the consequence of an improvement in public awareness of global vitamin D deficiency, its positive skeletal and extra-skeletal effects, as well as more intensive vitamin D supplementation due to the COVID-19 pandemic in the recent years.

## Introduction

Vitamin D is a fat-soluble group of vitamins that plays an important role in the human body. Its receptors were found in the nuclei and cell membranes of almost all human organs and tissues that, due to different genomic and non-genomic mechanisms, ensure the realization of its numerous skeletal and extra-skeletal effects. Vitamin D is not only an important regulator of calcium-phosphorus metabolism, which plays a great role in the prevention of rickets and osteomalacia, but also in a number of immune, endocrine, nervous, and cardiovascular systems diseases and some common types of cancer [1–4]. Vitamin D deficiency (VDD) is an increasing problem at a global level, and it has a great influence on the risk of many various diseases and mortality.

Nowadays, the data from numerous studies confirmed a high rate of VDD worldwide [5–10]; however, its frequency significantly varies. According to the current knowledge, the main reasons for VDD are insufficient exposure to sunlight in some populations due to geographic locations of some countries, in young people due to their lifestyle, and in older subjects due to a decreased vitamin D synthesis in the skin and absorption from the food. Also, dark skin pigmentation, the presence of diseases or conditions which have an important impact on vitamin D metabolism (obesity, malnutrition, kidney or liver failure, certain types of cancer, etc.), certain drug usage (glucocorticoids, anticonvulsants, cytostatics, etc.) are important factors for the VDD. Previous studies [8] demonstrated that the mean rate of the VDD in the world consists of about 37%, and it was the lowest in the USA (18%) in contrast to the countries of Europe (40%) and Africa (34%). Another study demonstrated that the rate of VDD in the Northern Europe population is lower (approximately 20%), and it was higher (30–60%) in Western, Southern, and Eastern European countries [9].

In 2011, the Institute of Medicine (IOM) and the Endocrine Society's Clinical Guidelines Subcommittee suggested evaluating the vitamin D status using the measurement of the total serum level of 25-hydroxyvitamin D (25(OH)D) and proposed the next ranges for vitamin D status: VDD (25(OH)D serum level ≤ 20 ng/mL or ≤ 50 nmol/L), vitamin D insufficiency (VDI: 21–29 ng/mL or > 50 and < 75 nmol/L) and normal vitamin D level (≥ 30 ng/mL or ≥ 75 nmol/L) [11].

Up to date, four Ukrainian epidemiological studies regarding vitamin D status were performed between 2011 and 2019 [12–15]. They established a high rate of VDD among the Ukrainian population regardless of age, gender, and region of residence. This finding and the wide implementation of vitamin D guideline for the Central Europe countries [16] raised the level of vitamin D prescriptions in Ukraine. During the last few years, COVID-19 pandemic became another important factor for the increase of vitamin D testing and supplementation in different countries in general and in Ukraine in particular. However, there are no data about the rate of VDD in Ukraine during the last years, which became the reason for the conducting this study.

## Methods

The research aimed to assess frequency of VDD and VDI in the Ukrainian population in 2016–2022 and to compare the results to the data of previous Ukrainian epidemiological studies.

### Study design

In the single-center study conducted by the State Institution "D. F. Chebotarev Institute of Gerontology of the National Academy of Sciences of Ukraine", we retrospectively analyzed 25(OH)D levels in blood serum of 8,758 adults aged from 20 to 99 years who, for various reasons, sought the measurement of (25(OH)D) in the Institute between 01 January 2016 and 31 December 2022. The sample was not drawn and analyzed only until March 2022 due to the Russian aggression in Ukraine in February and the impossibility of conducting the research.

### Population

Among 8,758 of the subjects whose data were available, 7,418 were selected for the analysis.

In the analysis, we included the data of the subjects of both sexes aged 20–99 years old which firstly in the definite years (2016–2022) measured 25(OH)D blood level. Supplementation of the vitamin D (200–4000 IU/d during the last 3 months) was allowed for inclusion in this study. Before the blood drawing, all participants signed informed consent for the use of their data for scientific studies according to the requirements of the Institute.

Exclusion criteria were the presence of clinically significant co-morbid pathologies with sub- and decompensation stages, diseases or conditions with a proven effect on vitamin D metabolism (liver, kidney failures, etc.), previous cancers, as well as vitamin D supplementation in the doses ≥ 4,000 IU/d at the time of examination or 3 months before it.

The analysis of the results was performed depending on sex, age (the groups were divided by decades), months, seasons, and years of the examination and vitamin D status. For the studying of the seasonal effects on 25(OH)D levels, samples had been grouped depending on the time of their collection into 4 seasons: winter (December-February), spring (March–May), summer (June–August), and autumn (September–November).

### 25(OH)D measurements

Vitamin D level (ng/mL) was measured by the value of 25(OH)D total (25-hydroxyvitamin D_2_ and 25-hydroxyvitamin D_3_) in blood serum. Venous blood sampling was drawn from 8:30 a.m. to 10:00 a.m. in the subjects fasting for at least 12 h. Blood samples were collected in vacutainer tubes with EDTA and gel, centrifuged, and separated, then, following cold chain principles, sent to the laboratory for measurement of 25(OH)D levels. It was assessed using the electrochemiluminescence method on Cobas^®^ e411 analyzer, (RocheDiagnostics^®^, Germany). This method allows to determine a 25(OH)D total in the range from 3.0 to 70.0 ng/mL. The sensitivity of the method consisted of 3.01 ng/mL, and the coefficient of variation is 7.5%. Samples with vitamin 25(OH)D concentration higher than the measurement range were manually diluted 1:2 and multiplied by the dilution factor.

The 25(OH)D level in blood serum was assessed according to the Central and Eastern European Expert Consensus [17] and was discriminated on the following ranges of serum 25(OH)D: concentration of < 20 ng/mL was considered as VDD, ≥ 20 ng/mL and < 30 ng/mL was considered as VDI. 25(OH)D, concentration of 30–50 ng/mL was considered as vitamin D sufficiency, > 50–60 ng/mL—as safe but not as a target level of vitamin D, > 60–100 ng/mL—as the area of uncertainty with potential benefits or risks, and the 25(OH)D concentration of > 100 ng/mL was considered as oversupply/vitamin D toxicity. For comparison of 25(OH)D level with data of other studies where it was presented in nmol/L) we used the coefficient *2.5.

### Statistical analysis

Statistical analysis was performed using STATISTICA 10 software (Serial Number: STA999K347150-W). For continuous variables, the results are presented as the mean value (M) and its standard deviation (M ± SD) in case of normal distribution or median and lower and upper quartiles (Me [25Q-75Q]) in case of the non-normal distribution of studied variables, for quantitative variables – in n (%). The continuous variables with normal distribution were compared using the Student test or One-way analysis of variance ANOVA (for comparison of 2 or more than 2 independent groups), others – using the Mann–Whitney or Kruskal–Wallis tests (for comparison of 2 or more than 2 independent groups, respectively). Differences in the distribution of the two samples were assessed using the χ^2^ test. The calculation of M ± SD in the case of the non-normal distribution of the variables was used for comparing our results with the data of other researchers. Differences between indices were considered significant when *p* < 0.05.

## Results

The females formed 88.3% (*n* = 6552) of all examined subjects and males, respectively, 11.7% (*n* = 866). The mean age of the subjects was 60.6 ± 13.8 years old (females were significantly older than males, respectively, 61.7 ± 13.0 and 52.5 ± 16.9 years, t = 18.9; *p* < 0.0001). 19.3% of the studied group confirmed the vitamin D supplementation during the last 3 months (200–4000 IU/d).

The mean level of the serum 25(OH)D in the total group consisted of 31.0 [22.3–41.1] ng/mL (M ± SD: 32.6 ± 14.5 ng/mL), with minimum and maximum values of 3.0 and 132.9 ng/mL, respectively. The analysis in the total group revealed that 3,089 subjects (41.6%) had an optimal (30–50 ng/mL) serum concentration of 25(OH)D, 2,023 persons (27.3%) had VDI, and 1,449 subjects had VDD (19.5%). A suboptimal and high serum concentration of 25(OH)D (50–100 ng/mL) in the total group was found in 551 and 293 persons, respectively (7.4 and 4.0%), 13 subjects (0.2%) had potentially toxic 25(OH)D concentrations (> 100 ng/mL).

In the total group, women had significantly higher 25(OH)D serum level (Me [25Q-75Q]: 31.2 [22.5–41.3] ng/mL; M ± SD: 32.8 ± 14.6 ng/mL) compared to the indices in men (Me [25Q-75Q]: 29,8 [21.0–39.8] ng/mL; M ± SD: 31.2 ± 14.2 ng/mL) (Z = 2.89; *p* < 0.004). However, the analysis in the age subgroups did not find any significant differences except for the group aged 60–69 years (Z = 3.88; *p* < 0.0001), where the 25(OH)D level in females was higher than in males.

We did not establish any significant differences in serum 25(OH) level depending on age groups in males (H = 12.3; *p* = 0.09), however, they were found in females (H = 82.8 *p* = 0.00001). The lowest 25(OH) level in women was found in the oldest age (90–99 years), and the highest one in the subjects aged 60–69 years old (Table 1). The level of 25(OH)D in persons aged 90–99 years was significantly lower than the corresponding indices in all younger age groups. In the 60–69 years old age group the level of 25(OH)D in women was significantly higher than parameters in the 30–39, 70–79, 80–89, and 90–99 years old age groups (Table 1).

**Table 1:** 25(OH)D level in blood serum of the subjects depending on age, ng/mL

Age groups, years	Total group		Men		Women	
	n	Me [25Q-75Q]	n	Me [25Q-75Q]	n	Me [25Q-75Q]
20–29	243	29.0 [20.2–39.1]*	82	27.6 [18.4–38.1]	161	30.0 [21.3–39.6]*
30–39	475	29.3 [21.6–39.2]*^#^	135	29.3 [21.0–39.8]	340	29.3 [22.0–39.1]*^#^
40–49	672	31.6 [22.6–42.0]*	179	31.4 [21.9–40.5]	493	31.7 [22.9–42.3]*
50–59	1554	31.6 [23.6–41.6]*	145	31.9 [23.7–42.3]	1409	31.5 [23.6–41.6]*
60–69	2517	32.1 [23.1–42.0]*	164	26.8 [20.9–36.6]	2353	32.5 [23.5–42.4]*
70–79	1485	29.8 [19.9–39.7]*^#^	124	32.3 [20.1–40.5]	1361	29.6 [19.9–39.6]*^#^
80–89	453	29.9 [18.8–40.5]*^#^	30	28.1 [15.5–38.5]	423	30.1 [18.9–40.7]*^#^
90–99	19	17.0 [8.2–28.9]^#^	7	28.9 [3.2–32.3]	12	14.5 [9.2–21.0]^#^
All Group	7418	31.0 [22.3–41.1]	866	29.8 [21.0–39.8]	6552	31.2 [22.5–41.3]

The difference between the indices was evaluated using the Mann–Whitney test; * – significant differences (*p* < 0.05) compared to the age group of 90–99 years; ^#^ – significant differences (*p* < 0.05) compared to the age group of 60–69 years

The analysis of the 25(OH)D level depending on the year of examination (2016–2022, Table 2) revealed a significant increase of the 25(OH)D level during the studied period (H = 572.1; *p* < 0.0001).

**Table 2:** The serum 25(OH)D level depending on the year of observation and sex, ng/mL

YYear	All subjects			Men		Women	
	n	Me [25Q-75Q]	M ± SD	n	Me [25Q-75Q]	n	Me [25Q-75Q]
2016	1028	25.1 [17.5–32.8]*	25.9 ± 11.6	120	24.4 [16.4–33.2]*	908	25.2[17.6–32.7]*
2017	1361	28.9 [19.8–37.3]*	29.1 ± 12.3	173	28.4 [20.2–38.9]	1188	28.9 [19.7–37.2]*
2018	1390	30.2 [21.7–40.3]*	31.5 ± 13.0	189	31.1 [20.3–40.4]	1201	30.3 [21.8–40.6]*
2019	1432	32.0 [23.2–42.2]*	33.3 ± 14.4	144	30.2 [21.4–42.4]	1288	31.9 [23.2–41.9]*
2020	786	36.8 [27.5–47.2]	38.1 ± 15.9	73	32.3 [22.6–42.4]	713	37.2 [28.0–47.8]
2021	805	35.0 [26.8–45.8]	37.2 ± 15.8	101	31.4 [24.2–39.8]	704	35.5 [27.0–46.4]
2022	616	36.0 [26.8–49.5]	38.9 ± 16.2	66	32.3 [22.8–42.1]	550	36.9 [27.1–50.3]

The difference between the indices was evaluated using the Mann–Whitney test; * – significant differences (*p* < 0.05) compared to the level in 2022

The mean annual serum 25(OH)D levels for 2022 was significantly higher compared to 2016, 2017, 2018, and 2019 years and did not differ from the indices in 2020 and 2021.

The analysis of the distribution of the subjects regarding the serum 25(OH)D level during the studied period confirmed the decrease in the VDD from 33.7% in 2016 to 9.3% in 2022 (Table 3). Also, during the last four years (2019–2022) we revealed the subjects with toxic vitamin D concentrations in the blood.

**Table 3:** Distribution of the subjects regarding to serum 25(OH)D level depending on the year of examination

Serum level of 25(OH)D, ng/mL	2016	2017	2018	2019	2020	2021	2022
< 20 (deficiency)	346 (33.7)	344 (25.3)	286 (20.6)	252 (17.6)	86 (10.9)	78 (9.7)	57(9.3)
20–29 (insufficiency)	341 (33.2)	392 (28.8)	387 (27.8)	378 (26.4)	162 (20.6)	214 (26.6)	149 (24.2)
30–50 (optimal values)	307 (29.9)	557 (40.9)	595 (42.8)	612 (42.7)	387 (49.2)	370 (46.0)	261 (42.4)
> 50–60 (suboptimal values)	25 (2.4)	55 (4.0)	92 (6.6)	135 (9.4)	90 (11.5)	84 (10.4)	70 (11.4)
> 60–100 (high concentration)	9 (0.9)	13 (1.0)	30 (2.2)	53 (3.7)	54 (6.9)	56 (7.0)	78 (12.7)
> 100 (toxic concentration)	0	0	0	2 (0.1)	7 (0.9)	3 (0.4)	1 (0.2)

We had found a significant effect of the month of blood collection on serum 25(OH)D levels (H = 94.9; *p* = 0.0001). The serum level of 25(OH)D was the highest in September and October and the lowest – in March (Table 4). The mean 25(OH)D level in March was lower than in other months except for February, other spring months (April and May), and two first summer months (June and July).

**Table 4:** The serum 25(OH)D level depending on the month of observation, ng/mL

Month	n	M ± SD	Me [25Q-75Q]
January	524	33.9 ± 14.7	31.2 [23.9–42.1]*
February	646	31.7 ± 16.3	29.4 [19.0–41.8]^#^
March	633	29.3 ± 14.4	27.7 [18.8–37.6]^#^
April	475	30.3 ± 13.9	29.7 [19.9–39.1]^#^
May	578	30.8 ± 14.8	29.3 [19.9–39.7]^#^
June	685	31.3 ± 13.5	30.4 [21.4–39.6]^#^
July	558	32.3 ± 12.8	31.0 [23.7–39.8]
August	590	33.6 ± 13.9	31.8 [24.5–41.4]*
September	714	34.7 ± 13.6	33.6 [25.7–42.6]*
October	707	34.9 ± 15.2	33.6 [24.0–42.8]*
November	708	32.9 ± 14.5	31.1 [22.7–41.9]*
December	600	34.4 ± 15.2	31.8 [23.5–44.0]*

The difference between the indices was evaluated using the Mann–Whitney test;* – significant differences (*p* < 0.05) compared to March; ^#^ – significant differences (*p* < 0.05) compared to October

Also, we found significant seasonal variations in serum 25(OH)D levels (H = 85.1 *p* = 0.0001). The median serum 25(OH)D level was the highest in autumn (33.0 [24.0–42.4] ng/mL) and was the lowest in spring (28.5 [19.7–38.7] ng/mL). The 25(OH)D concentration in winter and summer consisted of 30.7 [22.1–42.6] and 31.1 [23.1–40.3] ng/mL, respectively. The level of 25(OH)D in blood serum in spring was significantly different from the values in winter (Z = 5.92; *p* < 0.0001), summer (Z = 5.33; *p* < 0.0001), and autumn (Z = 9.14; *p* < 0.0001). The mean 25(OH)D level in autumn was significantly higher than the indices in winter (Z = 3.01; *p* < 0.02) and summer (Z = 3.70; *p* < 0.001).

**Fig. 1:**
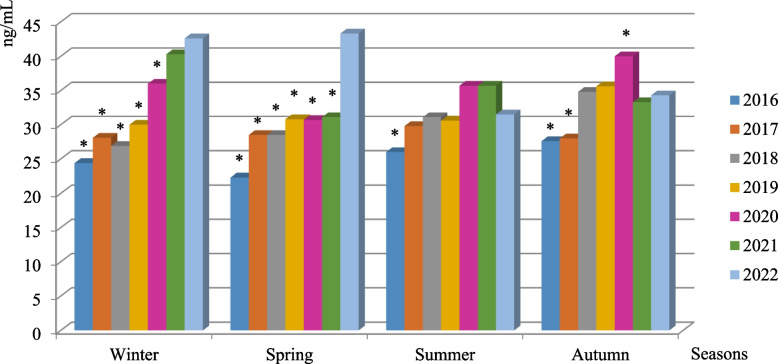
Serum 25(OH)D level depending on the seasons and years of examination. Note. Data are presented in Median value; the difference between the indices was evaluated using the Mann–Whitney test; * – significant differences (*p* < 0.01) compared to the indices in 2022 (same season) was evaluated

Analysis of the 25(OH)D level depending on year and season revealed that in the winter of 2022, the 25(OH)D level was significantly higher than the corresponding indices during the winter seasons of 2016–2020 (for all indices *p* < 0.001) without significant difference compared to the index in winter 2021. It was also higher in spring 2022 than the indices of the studies of other years. The mean 25(OH)D level did not differ significantly in summer depending on the year of observation for 2017–2022 years, although the index in 2016 was significantly lower than the ones in other years. During autumn, the 25(OH)D level also did not differ significantly in 2018, 2019, 2021, and 2022 years, only it was higher in the fall 2020 and lower in the same season in 2016 and 2017 years compared to the corresponding index in 2022 (Fig. 1).

## Discussion

Presently, it is well known that vitamin D gains importance worldwide for its crucial role in the development of its skeletal and numerous extra-skeletal effects. Many studies from different countries [5–10] had demonstrated the regional particularities of the VDD and the VDI depending on sex, age, and seasons. Current data demonstrate increased testing for 25(OH)D levels in the past decade [18], and the interest in vitamin D has increased in recent years due to the COVID-19 pandemic and its possible effects in the prevention of COVID-19-associated complications [19, 20].

The analysis of existing Ukrainian epidemiologic studies devoted to the investigation of the vitamin D status revealed four of them. The first one, published in 2011 [12] with the participation of adults from different regions of Ukraine showed a high level of the VDD (81.8%) and the VDI (13.6%). The authors demonstrated the increased VDD frequency in older age groups and the seasonal 25(OH)D variations. Later published Ukrainian epidemiological study [14] with the participation of children and adults with musculoskeletal pathology confirmed the decreased shares of the VDD and the VDI (37.3 and 30.6%, respectively) in 2017 compared to the data from the previous research. The results of two other regional Ukrainian studies performed in Bukovyna and Subcarpathia [13] and Transcarpathia [15] also confirmed the high shares of the VDD and the VDI with seasonal variation of 25(OH)D levels in children and adult populations. Despite the important finding, all these studies were performed before the COVID-19 pandemic, which increased interest in the vitamin D and its positive effects on human health that could have a significant effect on 25(OH)D levels and vitamin D supplementation. The data about the vitamin D status in Ukraine during the last years are absent which became the reason for this study's conduct.

Our study demonstrated higher serum levels of 25(OH)D in 2016–2022 (32.6 ± 14.5 ng/mL in the total group) compared to all previous Ukrainian studies (34.49 ± 0.53 nmol/L (equal to 13.80 ng/mL) [12], 21.6 ± 7.2 ng/mL [13], 26.2 ± 11.9 ng/mL [14], 22.67 ± 8.63 ng/mL [15], respectively) and the lowest share of VDD (19.9% in this study and 81.8% [12], 46.9% [13] and 37.4% [14] in the three other Ukrainian studies.

Previous Ukrainian studies revealed age-related features of serum levels of 25(OH)D but their fluctuations differed. The first Ukrainian study [12] demonstrated a significantly higher 25(OH)D level in the subjects aged 20–29 old, the study performed in Transcarpathia [15] confirmed the significantly higher 25(OH)D levels in children compared to adults with the highest level of VDD in the adults over 60 years. Our study also found a significant influence of age on the serum level of 25(OH)D with the lowest index in the subjects aged 90–99 years old and the highest one in the age group 60–69 years old, but only in females.

Our data confirmed the pronounced seasonal variations in serum 25(OH)D levels depending on the season of blood sampling demonstrated in other Ukrainian studies [12–15]. It is well known that winter and spring are the seasons with lower vitamin D levels compared to the indices in summer and autumn. In our research, the highest overall median serum 25(OH)D level was found in autumn, and the lowest one was in spring. However, a comparison of mean month fluctuations of the serum 25(OH)D level revealed the highest levels of 25(OH)D level in September and October in contrast to the results of our colleagues [14] which established the highest 25(OH)D concentration in August and September. The lowest 25(OH)D serum values were in February and March in the previous study [15] and in March in our research.

There are some reasons for the stark discrepancies in results between the performed studies. The first one is the method of 25(OH)D measurement. The first three Ukrainian studies [12–14] used the polyclonal Vitamin D assay (electrochemiluminescent method) on Elecsys 2010 analyzer (RocheDiagnostics®, Germany). However, during the last years, Roche Diagnostics® withdrew several lots of this assay due to a deterioration of conformity to the reference method for the reported results. Also, it was reported that polyclonal assays can lower the actual serum 25(OH) D levels [21]. In contrast to the previous studies, our colleagues and us [15] studied 25(OH)D levels using the Elecsys assay on analyzer Cobas® e411 (RocheDiagnostics®, German) with a newer generation kit for different analyzers.

The second reason for the established discrepancies is the year of observation and events that happened that year. Our analysis depending on the year of observation (2016–2022) confirms this hypothesis. The first Ukrainian study [12] revealed the lowest 25(OH)D level among all performed studies. The other research demonstrated higher serum 25(OH)D concentration [14] and our study established the significant influence of the examination year on 25(OH)D level (H = 475.5; *p* < 0.0001) with the highest share of VDD in 2016 (33.7%), less share in 2018 (20.6%), 2020 year (10.9%) and the lowest one in 2022 (9.3%). This increase during the last years can be related to the growing awareness about the VDD and the VDI in Ukraine and its positive skeletal and extraskeletal effects among both health professionals and patients. It is interesting that the 25(OH)D level in the total group in 2016 in our study (25.9 ng/mL) did not differ significantly from the results of our colleagues [14] published in 2017 (26.2 ng/mL) despite the use of different assays for the measurement of 25(OH)D level (Elecsys 2010 or Cobas® e411).

The important reason that can have influenced the growth of 25(OH)D level in the blood serum of the Ukrainians during the last years in Ukraine (2020–2021) can be the COVID-19 pandemic and increased public attention of the positive effects of vitamin D, in particular, in the respiratory tract infection prevention [19, 20] and decrease of COVID-19 severity and consequences [22]. Our results, possibly, can be explained by the increased consumption of vitamin D in prophylactic doses in order to protect against the COVID-19 infection and to prevent its severe course, especially in risk groups at the beginning of the pandemic. This hypothesis can confirm that the highest serum 25(OH)D level in autumn 2020 can be related to more intense vitamin D supplementation in the Ukrainian population during the first fall after the COVID-19 pandemic.

The other important reason that may influence annual 25(OH)D can be war in Ukraine which started with Russian aggression on 24.02.2022. We could not draw blood samples in March 2022 due to the impossibility to perform the investigation (repurposing of the Institute for other medical needs). Traditionally, this month demonstrates one of the lowest serum 25(OH)D concentrations and this fact can influence our results in spring 2022. Also, the decreased level of vitamin D supplementation due to the war in Ukraine may influence the mean 25(OH)D concentration in autumn 2022.

The comparison of our results with the data of neighboring countries [23–27] showed a higher mean annual level of 25(OH)D compared to the data from Hungary [23], Poland [24], Romania [25, 26], and Slovakia [27]. However, these results were published in 2013–2017 and this fact can influence study results. Also, the population and the methods of vitamin D measurement can influence stark discrepancies in results.

In the study of Hungarian men [23], the mean 25(OH)D level was 72.8 [11–185] nmol/L (equal to 29.12 ng/mL), the highest serum 25(OH)D levels were found in late summer (compared to the early autumn in our study), and lowest levels in late winter (compared to the early spring in our study). However, the Hungarian study included only relatively healthy men over 50 years of age that did not provide data on age-specific vitamin D status in the general population. Also, the authors measured plasma 25(OH)D level by high-pressure liquid chromatography (HPLC) using the Jasco HPLC system (Jasco, Tokyo, Japan) and Bio-Rad reagent kit (Bio-Rad Laboratories, Hercules, CA, USA) that differ from our study and can influence on the results.

The study from Poland [24] demonstrated the mean level of 25(OH)D of 18.0 ± 9.6 ng/mL in5 adult volunteers aged 15.6–89.8 years, however, the authors also used another method of 25(OH)D measuring (the Liaison XL DiaSorin system: CLIA method; DiaSorin, Saluggia, Italy). This study revealed the lower 25(OH)D levels in men and younger persons, but the collection of blood samples was performed in months with low solar activity (in spring) which can influence study results.

A large study of the vitamin D status from Romania with the participation of subjects with normal bone mineral density) [25] showed similar to our results of seasonal variations with the lowest 25(OH)D serum level in March and highest one in September. Since both levels in our study were higher than in the population of Romania, we can assume a more intensive intake of vitamin D in the Ukrainian population due to the COVID-19 pandemic. In the Romanian study, data from 2012 to 2016 were analyzed. If we take into account the data in Ukraine before the COVID-19 pandemic (in 2016), obtained results will be similar. Therefore, the highest levels in the Ukrainian population were found in August and September, and the lowest one – in February and March (data not presented in the results).

In another study from Romania [26] with the participation of adults without hyperparathyroidism, hypoparathyroidism, or low bone mass the mean 25(OH)D level was also lower than our results. However, the study used another vitamin D method (Liaison XL analyzer, DiaSorin, Saluggia, Italy) and was also performed before COVID-19 pandemic that have influence for discrepancies with our results.

The study performed in Slovakia [27] with participation of healthy medication-free volunteers and measurement of serum level of 25(OH)D_3_ in blood serum had shown that its mean level was higher than in our study. Only 15% of the subjects had 25(OH)D_3_ deficiency, 26% had insufficiency, and 59% – satisfactory level (> 30 ng/mL) of 25(OH)D_3_. However, direct comparison of our results is impossible due to the different study method (RIA method (25(OH)D3, Immuno Diagnostic system, Boldon, UK in Slovak study) and type of 25(OH)D (25(OH)D_3_ in Slovak study and 25(OH)D total in our one).

Today, our study is the largest and most recently analyzed sample for studying VDD and VDI in Ukraine. As a result, we found that 19.5% of the subjects had VDD (25(OH)D level below 20 ng/mL), 27.3% had VDI (> 20 to 30 ng/mL)) and 41.6% of the examined had an optimal vitamin D level (> 30–50 ng/mL). In addition, our study confirmed the pronounced significant seasonal variations in serum 25(OH)D demonstrated in early Ukrainian studies. Thus, the lowest level was found in spring (March), and the highest level at the beginning of autumn (September). Similar mean levels of 25(OH)D were obtained in countries located at the same latitude as Ukraine where a typically temperate climate with four seasons is found but only in pre-COVID pandemic years. The data during the COVID-19 pandemic in the neighboring populations are absent. A comparison of our results with the data of other Ukrainian studies performed previously demonstrated the decreased share of the VDD and the VDI during the last years that can reflect the greater vitamin D supplementation in the Ukrainian population due to the COVID-19 pandemic and increased knowledge about the positive vitamin D effect among the Ukrainians.

A limitation of this study was its design and sample (the vast majority of the study participants were from the central regions of Ukraine), although our institute provides consultative assistance to the subjects from all regions of the country. Additionally, our research did not evaluate the features of supplemental (duration, forms, etc.) vitamin D intake in prophylactic doses in the studied population that can influence on study results.

## Conclusion

The mean serum 25(OH)D level in Ukrainian adults aged from 20 to 99 years was 31.0 [22.3–41.1] ng/mL, the lowest level was in the age group 90–99 years old. No gender differences in the serum level of 25(OH)D were found, except the 25(OH)D level in the women aged 60–69 years old who had higher parameters compared to men.

41.6% of the subjects had an optimal (30–50 ng/mL) vitamin D level, 27.3% had VDI, 19.5% had a VDD. The suboptimal and high serum concentration of 25(OH)D (> 50–100 ng/mL) had 11.4% of the persons, 0.2% had potentially toxic 25(OH)D concentrations (> 100 ng/mL).

We established the increase of serum 25(OH)D from 2016 to 2021 with the highest values in 2020–2022. Seasonal 25(OH)D levels variations revealed the highest serum 25(OH)D levels in autumn (33.0 [24.0–42.4] ng/mL) and the lowest one in spring (28.5 [19.7–38.7] ng/mL), with the highest 25(OH)D level in September and the lowest ones in March.

The comparative analysis with the data from previous Ukrainian studies has demonstrated the decrease shares of VDD and VDI in 2020–2022 while preserving their age and seasonal characteristics, which may be associated with an increased awareness of the global vitamin D deficiency, its positive skeletal and extraskeletal impact in recent years, as well as more intensive vitamin D supplementation due to COVID-19 pandemic.

## Data Availability

All data are stored in the appropriate way and are available upon request to the corresponding author.

## References

[1] Zmijewski MA (2019). Vitamin D and Human Health. Int J Mol Sci.

[2] Bouillon R, Marcocci C, Carmeliet G, Bikle D, White JH, Dawson-Hughes B, Lips P, Munns CF, Lazaretti-Castro M, Giustina A (2019). Skeletal and extraskeletal actions of vitamin D: current evidence and outstanding questions. Endocr Rev.

[3] Grant WB, Boucher BJ, Pludowski P, Wimalawansa SJ (2022). The emerging evidence for non-skeletal health benefits of vitamin D supplementation in adults. Nat Rev Endocrinol.

[4] Bouillon R, Manousaki D, Rosen C, Trajanoska K, Rivadeneira F, Richards JB (2022). The health effects of vitamin D supplementation: evidence from human studies. Nat Rev Endocrinol.

[5] Cashman KD (2022). Global differences in vitamin D status and dietary intake: a review of the data. Endocr Connect.

[6] Roth DE, Abrams SA, Aloia J, Bergeron G, Bourassa MW, Brown KH, Calvo MS, Cashman KD, Combs G, De-Regil LM, Jefferds ME, Jones KS, Kapner H, Martineau AR, Neufeld LM, Schleicher RL, Thacher TD, Whiting SJ (2018). Global prevalence and disease burden of vitamin D deficiency: a roadmap for action in low- and middle-income countries. Ann N Y Acad Sci.

[7] Amrein K, Scherkl M, Hoffmann M, Neuwersch-Sommeregger S, Köstenberger M, Tmava Berisha A, Martucci G, Pilz S, Malle O (2020). Vitamin D deficiency 2.0: an update on the current status worldwide. Eur J Clin Nutr.

[8] Bouillon R (2020). Vitamin D status in Africa is worse than in other continents. Lancet Glob Health.

[9] Crowe FL, Jolly K, MacArthur C, Manaseki-Holland S, Gittoes N, Hewison M, Scragg R, Nirantharakumar K (2019). Trends in the incidence of testing for vitamin D deficiency in primary care in the UK: a retrospective analysis of The Health Improvement Network (THIN), 2005–2015. BMJ Open.

[10] Lips P, Cashman KD, Lamberg-Allardt C, Bischoff-Ferrari HA, Obermayer-Pietsch B, Bianchi ML, Stepan J, El-Hajj Fuleihan G, Bouillon R (2019). Current vitamin D status in European and Middle East countries and strategies to prevent vitamin D deficiency: a position statement of the European Calcified Tissue Society. Eur J Endocrinol.

[11] Holick MF, Binkley NC, Bischoff-Ferrari HA, Gordon CM, Hanley DA, Heaney RP, Murad MH, Weaver CM, Endocrine Society (2011). Evaluation, treatment, and prevention of vitamin D deficiency: an endocrine society clinical practice guideline. J Clin Endocrinol Metab.

[12] Povoroznyuk VV, Balatska NI, Muts VYA, Vdovina OA (2011). Deficiency and insufficiency of vitamin D in residents of Ukraine. Pain Joints Spine.

[13] Povoroznyuk VV, Pankiv IV. Deficiency and insufficiency of vitamin D in the inhabitants of Bukovyna and Prykarpattia. Int J Endocrinol. 2016;4:22–5 (Article in Ukrainian). Aссess: http://nbuv.gov.ua/UJRN/Mezh_2016_4_5.

[14] Povoroznyuk VV, Pludowski P, Holick M, Balatska NI, Dzerovych NI, Solonenko TYu, Ivanyk OS (2017). 25-hydroxyvitamin D levels, vitamin D deficiency and insufficiency in patients with bone and musculoskeletal disorders. Pain Joints Spine.

[15] Shchubelka K (2020). Vitamin D status in adults and children in Transcarpathia, Ukraine in 2019. BMC Nutr.

[16] Płudowski P, Karczmarewicz E, Bayer M, Carter G, Chlebna-Sokół D, Czech-Kowalska J, Dębski R, Decsi T, Dobrzańska A, Franek E, Głuszko P, Grant WB, Holick MF, Yankovskaya L, Konstantynowicz J, Książyk JB, Księżopolska-Orłowska K, Lewiński A, Litwin M, Lohner S, Lorenc RS, Lukaszkiewicz J, Marcinowska-Suchowierska E, Milewicz A, Misiorowski W, Nowicki M, Povoroznyuk V, Rozentryt P, Rudenka E, Shoenfeld Y, Socha P, Solnica B, Szalecki M, Tałałaj M, Varbiro S, Żmijewski MA (2013). Practical guidelines for the supplementation of vitamin D and the treatment of deficits in Central Europe – recommended vitamin D intakes in the general population and groups at risk of vitamin D deficiency. Endokrynol Pol.

[17] Pludowski P, Takacs I, Boyanov M, Belaya Z, Diaconu CC, Mokhort T, Zherdova N, Rasa I, Payer J, Pilz S (2022). Clinical practice in the prevention, diagnosis and treatment of vitamin D deficiency: a central and Eastern European expert consensus statement. Nutrients.

[18] Pilz S, Zittermann A, Trummer C, Theiler-Schwetz V, Lerchbaum E, Keppel MH, Grubler MR, Marz W, Pandis M (2019). Vitamin D testing and treatment: a narrative review of current evidence. Endocr Connect.

[19] Lanham-New SA, Webb AR, Cashman KD, Buttriss JL, Fallowfield JL, Masud T, Hewison M, Mathers JC, Kiely M, Welch AA, Ward KA, Magee P, Darling AL, Hill TR, Greig C, Smith CP, Murphy R, Leyland S, Bouillon R, Ray S, Kohlmeier M (2020). Vitamin D and SARS-CoV-2 virus/COVID-19 disease. BMJ Nutr Prev Health.

[20] Gibson-Moore H. Vitamin D: what’s new a year on from the COVID-19 outbreak? Nutr Bull. 2021;46:195–205. 10.1111/nbu.12499. 10.1111/nbu.12499 PMC8207114 34149314

[21] Connell AB, Jenkins N, Black M, Pasco JA, Kotowicz MA, Schneider HG (2011). Overreporting of vitamin D deficiency with the Roche Elecsys Vitamin D3 (25-OH) method. Pathology.

[22] Shah K, Varna VP, Sharma U, Mavalankar D (2022). Does vitamin D supplementation reduce COVID-19 severity? a systematic review. QJM.

[23] Bhattoa HP, Nagy E, More C, Kappelmayer J, Balogh A, Kalina E, Antal-Szalmas P (2013). Prevalence and seasonal variation of hypovitaminosis D and its relationship to bone metabolism in healthy Hungarian men over 50 years of age: the HunMen Study. Osteoporos Int.

[24] Płudowski P, Ducki C, Konstantynowicz J, Jaworski M. Vitamin D status in Poland. Pol Arch Med Wewn. 2016;126(7–8):530–9. 10.20452/pamw.3479. 10.20452/pamw.3479 27509842

[25] Niculescu DA, Capatina CAM, Dusceac R, Caragheorgheopol A, Ghemigian A, Poiana C (2017). Seasonal variation of serum vitamin D levels in Romania. Arch Osteoporos.

[26] Niculescu DA, Deacu LG, Caragheorgheopol A, Dusceac R, Procopiuc C, Petris R, Poiana C (2020). Seasonal periodicity of serum parathyroid hormone and its relation with vitamin D in Romania. Arch Osteoporos.

[27] Sebekova K, Krivosikova Z, Gajdos M, Podracka L (2016). Vitamin D status in apparently healthy medication-free Slovaks: association to blood pressure, body mass index, self-reported smoking status and physical activity. Bratisl Lek Listy.

